# A Speck of Bone That Saved a Limb

**DOI:** 10.4269/ajtmh.25-0239

**Published:** 2025-07-29

**Authors:** Hima Gopinath, Nidhima Aggarwal

**Affiliations:** ^1^ Department of Dermatology, All India Institute of Medical Sciences, Mangalagiri, India;; ^2^ Department of Microbiology, All India Institute of Medical Sciences, Mangalagiri, India

A 20-year-old college student from a south Indian city presented to me with a swollen right sole and foot with multiple discharging sinuses for the past 2 years. He had twice noted white grains discharged from the sinuses. He had been to several doctors and undergone numerous biopsies over the last 2 years. He had received sulfamethoxazole and trimethoprim for a month and multidrug therapy for tuberculosis for 3 months. Frequent physician visits and having an undiagnosed, progressively enlarging foot had affected his education. He decided to place his hopes on our hospital, a new tertiary care facility in the region. Image-guided biopsies and cultures had previously detected organisms in a few of our mycetoma patients, but they failed to yield any clues in this patient. We performed sequential overnight saline dressings to isolate grains but were unable to identify any.

With no clues on the etiological agent and no options for more advanced investigations, I asked my resident, Dr. Vijay Kiran, to try curetting the sinuses for any discharge or grains. Dr. Kiran was sincere in the quest for the elusive organism. The patient was enthusiastic and willing to tolerate the pain from curetting. We had used topical anesthetic cream to numb the patient’s skin, but pain was the least of his concerns. An hour later, my dedicated resident was ecstatic. He had found a white grain. He carefully rushed the precious grain to the microbiology department. Alas, the microbiology resident revealed that it was a speck of bone, not a grain. It was a disappointing day both for the patient and for us. The next day, the microbiology consultant got the serendipitous thought of examining the speck of bone. This thought was our patient’s silver lining as the speck of bone showed a lining of gram-positive, nonacid-fast, filamentous bacteria resembling *Actinomycetes*. I started the patient on five cycles of the Welsh regimen for actinomycetoma as there was bone involvement. The patient showed improvement, with reduced swelling, no further discharge from the sinuses within 3 months, and no recurrence of symptoms over the next year.

I face a myriad of challenges with mycetoma. Patients from poorer and marginalized sections of society often present with more advanced stages of mycetoma. Many of these patients are middle aged and work in agricultural fields. They manage the condition with pain medication readily available over the counter in India. Many received empirical treatments based only on clinical features. Many resort to short-term antimicrobial agents only when there is a disabling flare in discharging sinuses. A few patients were given the option of amputation in other centers. However, all of the patients who I have seen preferred to walk through their lives with a deformed foot rather than choose amputation.

One patient with actinomycetoma presented to us and several other hospitals once or twice a year for 5 years. Painful flare-ups triggered these visits. However, as soon as there was temporary relief from the pain with the treatment, he defaulted on the therapy and resumed his work. He had given up on the hope for a cure, accepted his deformed foot as a part of his life, and believed that he only needed to control the pain during flares. The prolonged duration of treatment and lack of dramatic changes in the initial phases contribute to patients defaulting on some existing effective therapies.

The light at the end of the mycetoma tunnel seems too far and bleak. Collaborating beyond every border is essential to help patients “step into” their lives.

